# Predicting psychiatric inpatient costs

**DOI:** 10.1007/s00127-015-1152-9

**Published:** 2015-12-18

**Authors:** Ramon Sabes-Figuera, Paul McCrone, Emese Csipke, Tom K. J. Craig, Diana Rose, Bina Sharma, Til Wykes

**Affiliations:** King’s Health Economics, Health Service and Population Research Department, Institute of Psychiatry, King’s College London, London, UK; Department of Psychology, Institute of Psychiatry, King’s College London, London, UK; Social Psychiatry, Health Service and Population Research Department, Institute of Psychiatry, King’s College London, London, UK; Service User Research Enterprise, Health Service and Population Research Department, Institute of Psychiatry, King’s College London, London, UK; P024 Institute of Psychiatry, Psychology and Neuroscience, De Crespigny Park, London, SE5 8AF UK

**Keywords:** Costs, Inpatient care, Economic evaluation, Service use

## Abstract

**Purpose:**

A large proportion of mental health costs is inpatient care but little is known about their variation between patients. The aim of this study was to measure and identify the predictors of costs of staff contacts and activities on inpatient wards.

**Method:**

Inpatients from psychiatric hospital wards in south London were interviewed in 2008 and 2009 and staff contacts and use of activities recorded over a week and costs calculated. Regression analyses identified predictors.

**Results:**

Of 334 participants, 78 % used activities and 90 % had staff contacts. However, 41 % reported no nurse contact. Mean staff contact and activity costs were £197 and £30 per week, respectively. Staff contact costs were inversely related to age, and activity costs were higher for patients with higher levels of education. Patient satisfaction was positively associated with both costs.

**Conclusions:**

The costs of self-reported staff contacts and use of activities account for a small amount of total inpatient costs. Patients with higher costs appeared to have higher levels of satisfaction.

## Introduction

An increased focus on providing care in community settings has taken place in developed countries in recent decades. Despite this trend, psychiatric hospital inpatient services remain an important element of the mental health care system. In 2010/11 in England, investment in inpatient care for working age adults was estimated to be £2 billion, representing 38 % of all direct investment for this population [1].

The care and interventions offered on inpatient wards need to be evaluated, just as any other health care service, in terms of costs and outcomes. Such evaluations require data on the use of services and related costs incurred by those using these services, and for inpatient care this cost has been usually obtained by multiplying the number of days spent in hospital by the unit cost per day. While in some circumstances this may be appropriate, it does not take into account the very likely variation between patients. For evaluations of inpatient interventions it would be more helpful to identify and cost all the care inputs received whilst on a ward. This distinction is important because, even if the length of stay of two patients is the same, the use of resources might be quite different depending on activities attended and the care received. Data on the amount of care received by inpatients in terms of staff contacts and activities attended would be informative for establishing if these wards have the therapeutic ethos that is required to fulfil the purpose of this level of care. Such care will inevitably vary between patients and identifying reasons for such variation is also important for service planning.

Previous literature suggests that only a limited amount of time is spent by patients in contact with health care professionals or engaged in therapeutic activities [8]. Most economic evaluations focus on a broad range of services and daily inpatient care costs are assumed constant across patients. This ignores the fact that patients on any particular date will require different levels of care and will recognise some care inputs as meaningful and others not. To our knowledge such analyses have not been previously reported.

This paper asks the following questions: (1) what amount of ‘meaningful’ care is received by patients on psychiatric wards in a large inner-city hospital in south London in 2008/9? (2) What is the cost of this care? and (3) What demographic and clinic characteristics can predict these costs?

## Method

This study is part of the “patient involvement in improving the evidence base on inpatient care” (PERCEIVE) research programme. PERCEIVE focussed on the therapeutic environment on inpatient psychiatric wards emphasising service user views and also taking feedback from staff. The programme took place in 17 acute mental health wards of a large service (the South London and Maudsley NHS Foundation Trust) providing mental health care in South London to a population of around 1.1 million people. Inpatients were eligible to be participants in PERCEIVE if they had been on a ward for a minimum of 7 days (with the exception of a triage ward where length of stay would be for up to 3 days), could communicate in English and had the ability to consent. Data for the analyses in this paper were collected between September 2008 and October 2009.

Bexley and Greenwich Research Ethics Committee granted approval for this study (Ref: 07/H0809/49).

### Measures

(1) Socio-demographic data: sex, age, ethnicity, level of education, marital status, and living and employment situation, (2) clinical information: diagnosis, whether the current admission to hospital was under the Mental Health Act (i.e. involuntary) and at what age the service user had their first contact with mental health services and time since their first psychiatric admission. (3) Functioning: global assessment of functioning (GAF) [2] scores were obtained for each service user. In addition, in order to explore the impact of service users’ perceptions on service use, a measure of their perceptions of the quality of acute inpatient care (VOICE) [3] was included. This 19-item self-report measure was developed using participatory methodology as part of the PERCEIVE programme. It has strong psychometric properties and is acceptable to inpatients. The VOICE score is calculated by summing the ratings across all 19 questions with each question being rated from 1 (strongly agree) to 6 (strongly disagree) and higher values indicate a poor perception of the quality of care.

Data on use of services were collected using a service use schedule, the CITRINE (client services receipt inventory—inpatient), developed as part of PERCEIVE. This tool is completed in a 5–10 min interview between a service user and a researcher or to be self-completed by service users. Its main objective is to obtain the number and location of therapeutic activities and number and duration of one-to-one staff contacts for each service user in the week prior to the interview that they consider to be meaningful. (A broad definition of ‘therapeutic activities’ is used. Essentially it includes activities such as ward meetings, practical training in crafts, and medication management advice.) It is adapted for each ward to reflect the differences in the range of activities provided [3].

### Service costs

The service use data obtained with the questionnaire were combined with appropriate unit costs for 2007/8 obtained from national sources for care professionals [4]. Similar data were not available for the unit cost of therapeutic activities, and these were therefore calculated specifically for the study. Staff members responsible for organising activities were asked to provide details on the session duration, preparation required, staff involved and materials for each activity provided on each ward. These data were combined with unit costs for these resources and with information on the average number of service users attending each activity to obtain an estimated individual cost for each group activity. Activities were subsequently classified into categories according to their type and cost.

### Statistical analysis

Multiple regression models were constructed to identify factors that explained variations in costs. Dependent variables were the cost of one-to-one care contacts and costs of therapeutic activities. A simple regression model for each potential independent variable was run and those variables with statistical significance for any of the two dependent variables were included in the final model. All selected variables were entered in a single block in a fixed effects regression to take account of data being obtained from service users on 17 different wards. Cost data are often positively skewed and this may result in regression residuals that are similarly skewed, which is a violation of the assumptions underlying the linear regression model. Consequently non-parametric bootstrapping methods were used [5]. Bootstrapping involves resampling with replacement from the original data a sufficiently large number of times so that the population from which the sample is drawn can be approximated. Here, 5000 samples were automatically generated and bootstrapped 95 % confidence intervals were generated.

## Results

Of those service users who fulfilled the inclusion criteria, 62 % agreed to participate in the study. Service use data were available for 402 service users. However, some values for socio-demographic and clinical variables were missing for some participants or their length of stay was less than 7 days. It was decided to include in the analysis only participants with complete data, and the final sample size was 334. It was found that those with incomplete data had lower GAF functioning scores (35.6) than those included (39.8) (*p* < 0.01).

The characteristics of the sample are shown in Table 1. Two-thirds had a diagnosis of schizophrenia or bipolar disorder with most other patients having a primary diagnosis of depression, drug-related disorder or personality disorder. Around two-thirds also had been admitted under the Mental Health Act. The average length of stay at the point of assessment was almost 7 weeks, but with a wide range. The GAF scores indicate a high level of disability. As stated above, the VOICE was designed, so higher scores reflected lower quality of care. Therefore, those service users with a score of 38 or lower felt that the quality of care received was good. Alternatively, those with scores of 95, and higher, perceived that inpatient care is of low quality. In our sample, 21 % had a VOICE score of 38 or lower and 5 % had a score of 95 and higher. Therefore, most of the sample felt that the quality of care was neither good nor bad.

**Table 1:** Sample characteristics (*n* = 334)

Characteristic	*N* (%)
Female	143 (42.8)
Non-white ethnicity	176 (52.7)
12 or more years of education	131 (39.2)
Schizophrenia or bipolar disorder	218 (65.3)
Current admission to hospital under the Mental Health Act	216 (64.7)
Current admission is first admission	68 (20.4)

	Mean (SD)	Max	Min
Age (years)	40.0 (12.8)	75	18
VOICE scale score	54.8 (19.5)	114	19
GAF symptoms	42.4 (14.3)	80.0	1
GAF functioning	39.8 (10.9)	81.0	11
Length of stay at assessment (days)	48.4 (94.0)	1261	7

Table 2 summarises the data collected with the CITRINE instrument, providing information on the number of therapeutic activities attended and number and type of one-to-one care contacts reported by participants during the period of 7 days. Almost 10 % of the sample stated that they had not had any one-to-one contacts and more than one-fifth reported that they had not attended any activities. The average number of both one-to-one care contacts for the whole sample was well less than one-per day (Table 2).

**Table 2:** Therapeutic activities and one-to-one contacts in past week

	*N* (%)	Mean (SD)	Median	Max	Min
Therapeutic activities attended	262 (78.4)	3.9 (4.7)	2	25	0
One to one contacts with care staff	301 (90.1)	5.6 (5.4)	4	43	0

Type of one-to-one contacts	*N* (%)	Mean (SD) contacts for those in receipt	Mean (SD) duration for those in receipt (mins)
Nursing staff	198 (59.3)	4.1 (3.8)	16.8 (19.1)
Psychiatrist	247 (74.0)	1.6 (1.0)	18.6 (14.5)
Other doctor	84 (25.1)	1.7 (1.3)	16.2 (14.3)
Occupational therapist	68 (20.4)	2.6 (2.9)	21.8 (24.3)
Care coordinator	91 (27.2)	1.6 (1.4)	23.4 (25.3)
Other care staff	110 (32.9)	1.9 (2.2)	36.8 (32.7)

More than 40 % of participants reported no contacts with a member of nursing staff in the past week and for those who did have contact, their frequency was slightly higher than one contact every 2 days (Table 2). Participants with no nursing contacts were not more likely to have contact with other staff members (and so this does not indicate a problem of distinguishing between care staff). The number of participants who reported contacts with the other types of care staff was low, except for psychiatrists where almost three quarters of the sample had a weekly average of 1.6 contacts.

The service use data were combined with the unit costs for 2008 (see “Appendix Table 5”) to obtain the costs of therapeutic group activities and one-to-one care contacts during the previous week. There was significant ∞variation within the different group activities in the cost per participant attendance. This variation is the result of three factors: first, the different qualifications and number of professionals involved; second, the duration of the activities and the time necessary for their preparation; and finally, the number of participants who attended each activity. This number varied from 2 to 14.

Table 3 shows the average cost of group activities and one-to-one care contacts over the past week for the 334 participants. Costs were highest for time with a psychiatrist and accounted for more than half of the cost of care contacts. The cost of group activities represented a small proportion of the total cost as did contacts with nursing staff. If the total figure, cost of group activities and one-to-one care contacts, for the one-week period is translated to a cost per day, the value is slightly more than £30 per day. There is substantial variation between individuals, as reflected by the standard deviations.

**Table 3:** Cost of services and activities in past week (2007/8 £s)

Service/activity	Mean	SD	% of total
Nursing staff	29.3	69.2	12.9
Psychiatrist	109.0	131.0	48.1
Other doctor	17.4	45.0	7.7
Occupational therapist	6.5	21.0	2.9
Care coordinator	15.2	48.6	6.7
Other care staff	19.6	52.6	8.7
All one to one contacts	197.0	196.3	86.9
Therapeutic activities	29.7	38.8	13.1
Total cost	226.7	206.1	100.0

The regression analysis of the variation in costs of one-to-one contacts (Table 4) showed that older participants had lower costs. A higher score on the VOICE instrument was related to lower cost, indicating that participants with a worse perception of inpatient care also use fewer inpatient resources. For instance, a decrease in the VOICE score of 10 points would imply, assuming other variables are held constant, an increase in costs of £11 per week. There were significant cost differences between inpatient wards. Regarding the activities model, the VOICE score was again statistically significantly associated with cost (Table 4). Service users with a higher level of education had higher activity costs. (Subsequent analyses revealed this to be caused by more engagement with activities rather than use of higher cost activities.) The results of the two models also show the positive relationship between the two types of cost.

**Table 4:** Regression of one-to-one contact costs and activity costs in past week on demographic and clinical characteristics (clustering for ward)

Variable	Model of contacts cost			Model of activities cost		
	B	SE	95 % CI	B	SE	95 % CI
Age (years)	–1.75	0.66	–3.23 to –0.64	–0.15	0.16	–0.44 to 0.18
Gender (female vs male)	7.28	30.96	–74.01 to 48.65	9.36	6.32	–0.90 to 23.87
Ethnicity (non-white vs white)	–42.53	22.90	–84.85 to 4.31	0.84	2.43	–2.98 to 6.50
First admission (yes vs no)	34.18	28.86	–10.52 to 99.91	1.92	8.03	–15.90 to 15.38
Education (12 or more years vs <12)	46.99	21.10	–0.85 to 81.32	6.89	5.49	0.62 to 21.88
Diagnosis (schizophrenia and bipolar vs other)	3.24	18.13	–32.17 to 39.01	–2.95	5.99	–19.57 to 2.48
GAF symptoms	–0.55	0.76	–1.96 to 1.04	–0.27	0.18	–0.65 to 0.02
GAF functioning	0.47	1.07	–1.99 to 2.23	0.12	0.24	–0.21 to 0.70
VOICE (pro–rated if ≥16 items answered)	–1.13	0.33	–1.72 to –0.44	–0.21	0.11	–0.47 to –0.05
Length of stay (at assessment)	–0.12	0.14	–0.47 to 0.02	0.03	0.03	0.00 to 0.10
Activities cost	0.51	0.36	0.03 to 1.47	–	–	–
Contacts cost	–	–	–	0.02	0.01	0.00 to 0.04
Ward variable	*F* (16,306) = 2.66, *p* = 0.001			*F* (16,306) = 5.55, *p* < 0.001		

Costs in 2007/8 £s

## Discussion

This is the first study to present a detailed analysis of the cost of care contacts and activities on adult psychiatric inpatient wards. The results show that service users report low levels of contacts with staff members. It was striking that 40 % did not report contact with nursing staff. The results for therapeutic activities attended do not indicate that the low level of contacts with staff was compensated by more attendances. We have estimated that the cost of care contacts and therapeutic activities is around £30 per day, which is just slightly higher than 10 % of the average cost per bed day £288 for 2008/9 [6]. The latter figure includes the costs of other resources not considered in the present study such as cleaning maintenance, food, laundry, drugs, other treatments, etc. Crucially, the figures here do not include staff time that is not spent in contact with patients. In relation specifically to nursing staff costs, Bowers and Flood analysed expenditure data (i.e. a ‘top-down’ approach including non-contact time) for 136 wards, and they found a cost per bed day equal to £90 [7]. While in the current study we found that the daily cost of nursing staff contacts was £4.20. The difference could be partially caused by an under-reporting by service users of any contacts, and specifically nursing staff contacts. However, a review of studies that measured nursing and patient activity and interaction on psychiatric inpatient wards [8] found proportions of staff time spent in direct contact with patients (24–48 %) and/or providing specific therapeutic interventions (4–20 %). This indicates that the difference between top-down and ‘bottom-up’ (as here) calculated costs is probably not due to under reporting in the latter but due to low levels of activity.

High scores on the VOICE questionnaire, representing low satisfaction, were related to low costs of staff contacts and activities. However, the direction of the association is not clear; it is possible that attending fewer activities and having fewer contacts with staff is a result of a worse perception of care received or low levels of care received may lead to poor perceptions of care. In relation to socio-demographic characteristics of the patients, age was inversely related to staff contact costs while service users with more than 12 years of education had activity costs 23 % higher than those with fewer years of education. Differing results have been found on the impact of socio-demographic characteristics on mental health care costs [9]. Nevertheless, it is interesting than McCrone et al. found that in a sample of individuals with severe mental illness, older people and those with lower levels of education had higher costs of community mental care [10]. The age effect is the opposite of what was found here and may be related to other factors e.g. physical health in the elderly and/or chronicity relating to poor function. Also it should be recognised that these costs may be influenced by the structure of services; staff are present on inpatient wards (i.e. they are available) whereas the in the community it may be more complex to arrange a contact.

These findings need to be considered taking account of the limitations of the study. First, a relevant proportion of inpatients, almost 40 %, refused to participate in the study. It was not possible to collect information on demographic or clinical characteristics for this group of patients, therefore preventing a comparison with those who agreed to participate. This analysis would have allowed us to establish any significant difference in these variables that would indicate a possible different pattern in their use of services. Second, limitations of the instrument used to collect the data, the CITRINE questionnaire, need to be considered. These have been already discussed [3], specifically the problems with information on nursing staff contacts, where the bigger variation between sources were found. Nevertheless, alternative sources of information, registers/electronic databases and observational studies, are not free of problems. Further validation of the measure would be useful and this could come through accessing service use data from staff and carers. However, the focus here was on contacts considered meaningful by service users and it is difficult to collect such information from alternative sources.

The low costs found reflect low levels of reported interaction between care professionals and inpatients. This is concerning because it has been established that in inpatient care outcomes are associated to the level of attention patients received from staff [11]. The relationship found in this study between costs and service user’s views of the quality of care, measured by the VOICE instrument, emphasises these previous findings.

## Appendix

See Table 5.

**Table 5:** Unit costs of services and activities

Care professional	Cost per hour of patient contact (2007/8 £s)^a^
Psychiatrist	316
Psychologist	72
Social worker	89
Pharmacist	63
Occupational therapist	43
Nurse	43
Counsellor	40
Health care assistant	23
Advocate, volunteer	23
Activities coordinator	20

Activities	Cost per attendance (2007/8 £s)^b^
Community meeting	2.5
Bingo, current affairs group, feeling good group, games/quiz group, information trolley	4.8
Coping with stigma, gentle exercise, hearing voices group, narrative expression, sleep hygiene	7.0
Community outing, film club or film night, gardening group, healthy breakfast cooking, men’s group, sleep hygiene, women’s group	7.6
Arts and crafts	9.6
Building a compelling future group, CD DJ mixing, communication group, computer/internet access group, health promotion group, healthy eating group, healthy living group, IT skills group, learning how to cope group, Music group, planning your future group, pottery group, Tai Chi group, textiles group, vocational group, woodwork group, yoga group	10.0
Belly dancing, clinical exercise group, dance and movement therapy, exercise group, feeling good/reflexology/massage, music therapy group, reflexology group, religious or spiritual group	11.4
Art therapy group	11.6
Group therapy	13.3
Creative writing group, CRT, go to the gym, go to the swimming pool, medication group, remotivation process, staying well, swimming group	14.3
Relaxation group	15.9
Baking group, community visit, cooking group, daily planning meeting, drama therapy, go to the chapel, walking group	16.9
Complaints clinic	38.8
Cooking session	39.7

^a^Obtained or derived from Curtis^4^

^b^Calculated using ward data on activities and Curtis^4^
